# Prevalence of Acne and Its Association With Increased Body Mass Index Among Adolescent Schoolchildren in Northern Sudan

**DOI:** 10.1002/hsr2.71264

**Published:** 2025-09-23

**Authors:** Iqra Magsi, Muhammad Umar

**Affiliations:** ^1^ Liaquat University of Medical and Health Sciences Jamshoro Sindh Pakistan; ^2^ Khairpur Medical College Khairpur Mirs Sindh Pakistan

## Transparency Statement

Ms. Iqra Magsi affirms that this manuscript is an honest, accurate, and transparent account of the study being reported; that no important aspects of the study have been omitted; and that any discrepancies from the study as planned (and, if relevant, registered) have been explained.

Dear Editor,

I read the article “Prevalence of Acne and Its Association With Increased Body Mass Index Among Adolescent Schoolchildren in Northern Sudan” by Alotaibi et al., with great interest. This study addresses a significant public health concern by highlighting the prevalence of acne among adolescents and its potential association with a higher body mass index (BMI) [1]. Although the authors provide a novel approach, several methodological and analytical limitations should be taken into account to enhance future research in this area.

One primary limitation of the study is its cross‐sectional design, which limits the ability to establish causality between BMI and acne prevalence. A longitudinal study following adolescents over time would provide stronger evidence regarding the potential causal relationship between these variables [2]. Additionally, the limited sample size of 384 adolescents may not fully represent the broader Sudanese adolescent population reducing the generalizability of the findings. A larger and more diverse sample would enhance the statistical power of the study.

The selection of participants from only four schools in one locality raises concerns about selection bias, as it may not capture the full spectrum of socioeconomic and environmental factors affecting adolescent acne. Furthermore, the absence of a control group without acne limits the ability to determine whether BMI is a direct risk factor or merely correlated with other underlying conditions.

Statistically, while multivariate binary regression was performed, the study does not fully account for confounding variables such as hormonal fluctuations, dietary habits, genetic predisposition, and stress levels, all of which significantly impact both BMI and acne risk [3, 4]. Future research should incorporate meta‐regression techniques to more effectively evaluate the underlying trends and interactions among these factors.

Biologically, the study suggests an association between BMI and acne but does not include hormonal assessments, which are crucial for confirming the role of androgens, insulin, and insulin‐like growth factor‐1 (IGF‐1) in acne pathogenesis [5]. Measuring these biomarkers would strengthen the study′s conclusions. Similarly, nutritional assessments would improve the findings. Moreover, the study overlooks environmental factors such as pollution, stress, and skincare habits, which may contribute to acne prevalence. Exploring these influences could provide a more comprehensive understanding of the issue.

To advance research in this field, I recommend that future studies adopt longitudinal designs to establish a causal relationship between BMI and acne. Additionally, interdisciplinary collaboration among dermatologists, endocrinologists, and nutritionists should be integrated to enhance risk factor analysis. Utilizing objective biomarkers such as skin analyzers, hormonal assays, and inflammatory markers will also contribute to more precise data collection.

## Author Contributions

**Iqra Magsi:** conceptualization, investigation, writing – original draft, methodology, writing – review and editing, data curation. **Muhammad Umar:** supervision, project administration, formal analysis, writing – review and editing.

## Disclosure

“All authors have read and approved the final version of the manuscript Ms Iqra magsi had full access to all of the data in this study and takes complete responsibility for the integrity of the data and the accuracy of the data analysis.”

## Conflicts of Interest

The authors declare no conflicts of interest.

## Data Availability

Data sharing not applicable to this article as no datasets were generated or analyzed during the current study. No data is associated with the article or all data underlying the results presented are available in the article and freely available online to use.
